# A rare cause of diffuse alveolar hemorrhage in a pediatric patient: thigh localization of INI1-deficient epithelioid sarcoma

**DOI:** 10.1186/s13023-025-03956-1

**Published:** 2025-08-19

**Authors:** Xiaolei Tang, Zhipeng Zhao, Xingfeng Yao, Chunju Zhou, Shuai Gong, Haiming Yang

**Affiliations:** 1 Department of Respiratory Medicine, Beijing Children’s Hospital, Capital Medical University, National Center for Children’s Health, No.56 Nanlishi Road, Beijing, China; 2 Department of Pathology, Beijing Children’s Hospital, Capital Medical University, National Center for Children’s Health, Beijing, China; 3 Department of Neurology, Beijing Children’s Hospital, Capital Medical University, National Center for Children’s Health, Beijing, China; 4 Department of Respiratory Diseases, Pediatric Research Institute of Xinjang Uygur Autonomous Region, Children’s Hospital of Xinjang Uygur Autonomous Region, Xinjiang Hospital of Beijing Children’s Hospital, The Seventh People’s Hospital of Xinjiang Uygur Autonomous Region, Urumqi, China

**Keywords:** Epithelioid sarcoma, Diffuse alveolar hemorrhage, Intracranial hemorrhage, Pediatric, INI1 deficiency

## Abstract

**Background:**

Diffuse alveolar hemorrhage (DAH) is a group of rare but life-threatening conditions characterized by bleeding into the alveolar spaces, often associated with various etiologies. Epithelioid sarcoma (ES) is a rare and aggressive soft tissue sarcoma that has never been reported to be associated with DAH.

**Methods:**

We report a rare case of ES in a pediatric patient presenting with DAH and intracranial hemorrhagic lesions. Diagnostic evaluations included high-resolution chest CT (HRCT), bronchoscopy, brain MRI, lung biopsy, Positron emission tomography - computed tomography (PET-CT), and biopsy of a mass in the right thigh. Fluorescence in situ hybridization was performed to detect SMARCB1 (INI1) gene deletion.

**Results:**

A 13-year-old male presented with anemia and later developed hemoptysis with a decreased hemoglobin level. HRCT revealed bilateral ground-glass opacities consistent with DAH. Extensive autoimmune and infectious workups were all negative. Brain MRI demonstrated microhemorrhages. Despite corticosteroid therapy, the patient’s condition worsened. PET-CT identified a hypermetabolic soft tissue mass in the right thigh, which on histopathological examination was confirmed as INI1-deficient ES. FISH analysis confirmed INI1 deletion.

**Conclusion:**

This case highlights the diagnostic challenges of INI1-deficient ES presenting with DAH and underscores the importance of considering malignancy in pediatric patients with atypical DAH. Multidisciplinary approaches, including advanced imaging and molecular diagnostics, are crucial for accurate diagnosis and management.

## Background

Diffuse alveolar hemorrhage (DAH) is a rare clinical syndrome characterized by the accumulation of red blood cells in the alveolar spaces. This condition typically manifests as a triad of clinical features: hemoptysis, anemia, and bilateral diffuse ground-glass opacities on imaging studies. Although DAH is predominantly associated with autoimmune disorders (e.g., systemic vasculitis) and infectious processes, malignancy-associated DAH represents an exceptionally rare entity that often poses significant diagnostic challenges.

Epithelioid sarcoma (ES), a rare and highly aggressive soft tissue neoplasm, accounts for less than 1% of all soft tissue malignancies [1]. Pathologically, ES is characterized by the loss of INI1 (SMARCB1) expression, a critical tumor suppressor gene involved in chromatin remodeling processes. Clinically, ES typically presents as an indolent, painless mass; however, it exhibits a high propensity for local recurrence and systemic metastasis. Pediatric cases are particularly rare and often associated with a more aggressive clinical course [2]. While pulmonary metastases have been reported in ES, there are no previous reports of ES associated with DAH.

We herein report a diagnostically challenging case of INI1-deficient epithelioid sarcoma localized in the right thigh of a 13-year-old male patient who presented with DAH and concurrent intracranial hemorrhagic lesions. This case highlights the diagnostic complexities of malignancy-related DAH in pediatric patients and underscores the importance of considering neoplastic etiologies in cases of treatment-refractory DAH.

## Materials and methods

### Fluorescence in situ hybridization (FISH) analysis

FISH was performed on the soft tissue mass using a SMARCB1 (22q11) gene deletion probe (GSP SMARCB1 / GSP 22q12, Code: F.01291-01). Tissue sections were deparaffinized, pretreated with protease, and hybridized with the probe according to the manufacturer’s instructions. After hybridization, the slides were washed to remove unbound probes, and nuclei were counterstained with 4’,6-diamidino-2-phenylindole (DAPI). Fluorescence signals were visualized and analyzed using a fluorescence microscope equipped with appropriate filters. The deletion of the SMARCB1 gene was confirmed by the absence of fluorescence signals in tumor cells, while normal cells served as internal controls and exhibited two distinct signals.

### Positron emission tomography - computed tomography (PET-CT) imaging

The patient underwent whole-body PET-CT after fasting for 6 h (blood glucose: 4.9 mmol/L). 18 F-fluorodeoxyglucose (FDG) was intravenously administered, and imaging was performed 60 min post-injection. Emission and transmission scans were acquired from the head to the mid-thigh, and images were reconstructed using iterative algorithms. Standardized uptake values (SUV) were calculated for regions of interest.

## Results

### Case presentation

A 13-year-old male presented with a two-week history of dizziness and fatigue, accompanied by clinical signs of anemia. The patient denied fever, cough, headache, or other systemic symptoms. Physical examination revealed pallor but no evidence of digital clubbing, arthritis, myasthenia, or meningeal/pathological signs. The patient had no significant past medical history and no family history of hematologic disorders, rheumatic diseases, or malignancies.

Initial laboratory investigations revealed anemia with a hemoglobin level of 92 g/L, while white blood cell count, platelet count, and C-reactive protein levels were within normal ranges. A comprehensive hematologic workup, including coagulation studies, serum iron, total iron-binding capacity, vitamin B12, direct Coombs test, and hemoglobin electrophoresis, yielded normal results.

**Fig. 1:**
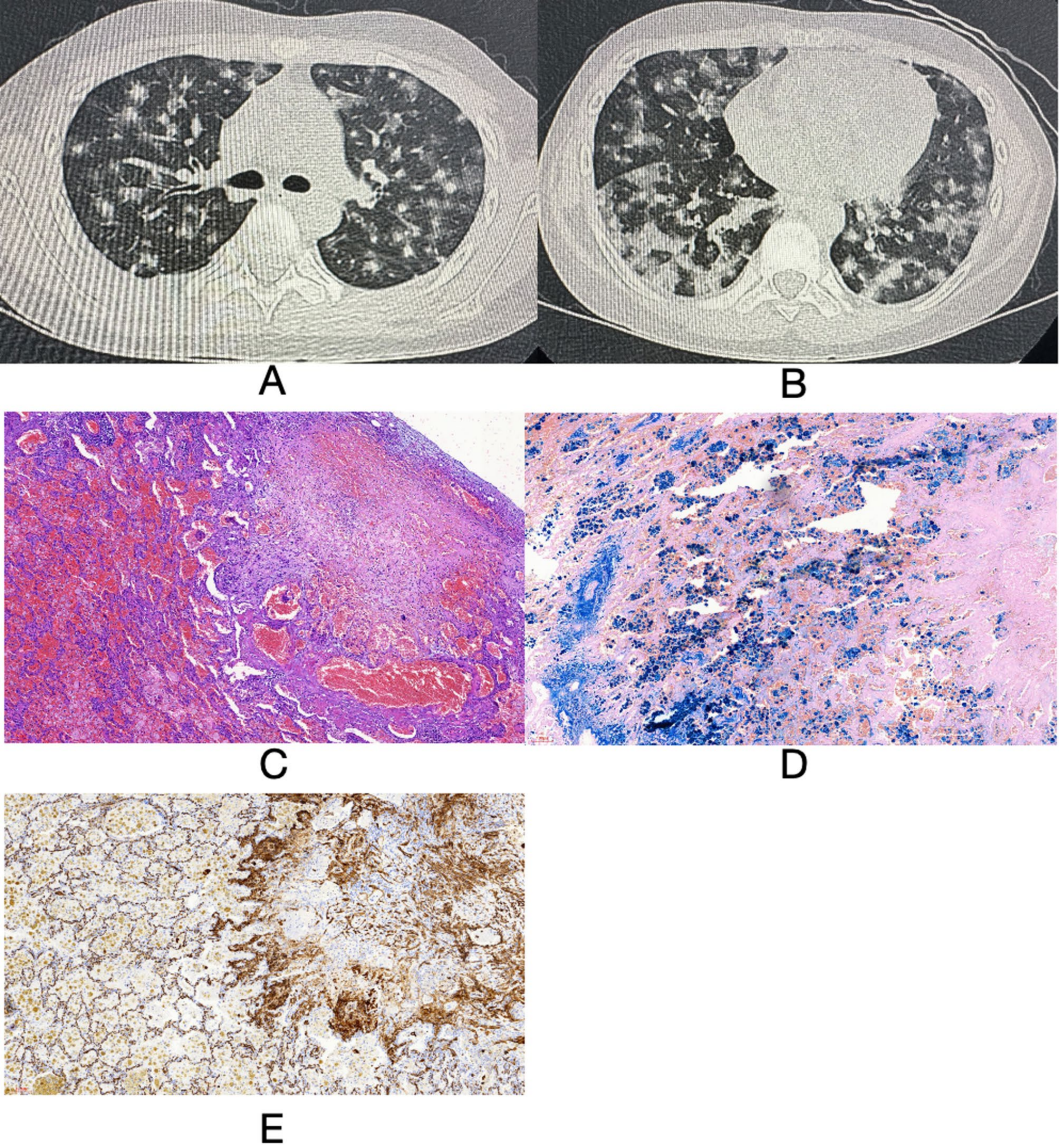
**A** and **B**: High-resolution chest CT (HRCT) demonstrated extensive bilateral nodular ground-glass opacities in both lungs. **C**: Histopathological examination of lung tissue (×10 magnification) revealed alveolar expansion with associated type II pneumocyte hyperplasia. The alveolar spaces contained abundant hemosiderin-laden macrophages. The alveolar septa exhibited mild thickening with infiltration of scattered lymphocytes and histiocytes. Vascular examination revealed mild degenerative changes in small vessel walls, with no significant perivascular inflammatory cell infiltration. Focal hemorrhagic necrosis and myofibroblast proliferation were observed, accompanied by infiltrating lymphocytes and plasma cells in the surrounding tissue. **D**: Prussian Blue staining of lung tissue (×10 magnification) showed abundant blue-stained hemosiderin-laden macrophages within the alveolar spaces. **E**: CD34 immunohistochemical staining of lung tissue (×10 magnification) demonstrated CD34-positive vascular endothelial cells

Two weeks after the initial presentation, the patient developed hemoptysis, and his hemoglobin level decreased to 71 g/L. High-resolution chest CT (HRCT) scan demonstrated extensive bilateral nodular ground-glass opacities (Fig. 1A and B). Bronchoscopy revealed diffuse endobronchial bleeding. Cytological analysis of bronchoalveolar lavage fluid (BALF) showed numerous hemosiderin-laden macrophages, consistent with diffuse alveolar hemorrhage (DAH).

A comprehensive etiologic work-up was undertaken. Extensive autoantibody panels were negative and complement levels were within normal limits. Immunoglobulin (Ig) assays revealed mildly decreased IgG, with normal IgA, IgM, and IgE. Lymphocyte subset analysis demonstrated abnormal absolute counts of T and B lymphocytes, and natural killer cells. Serum cytokine analysis revealed elevated interleukin (IL)-4 and interferon (IFN)-γ whereas tumor markers were noncontributory. Imaging studies, including sinus radiography, echocardiography, abdominal CT, and abdominal ultrasound, revealed no abnormalities. Pathogen detection via next-generation sequencing of BALF was positive for *Streptococcus pneumoniae*. The bone marrow aspirate revealed hypercellular marrow with marked erythroid hyperplasia (See Table 1 for details).

**Table 1:** Laboratory and imaging findings

	Test	Result
Autoimmune Serology	ANA, ds-DNA, ENA, ANCA, CCP, RF and aCL	Negative
Immunoglobulins	IgG	7.4 mg/L (mildly ↓)
	IgA, IgM and IgE	Normal
Complements	C3 and C4	Normal
Lymphocyte Subsets	Total T lymphocytes	689 /µL (↓)
	CD4 + lymphocytes	233 /µL (↓)
	CD8 + lymphocytes	405 /µL (↓)
	B lymphocytes	502 /µL (↑)
	NK cells	94 /µL (↓)
Serum Cytokines	IL-4	44.8 pg/mL (↑)
	IFN-γ	27 pg/mL (↑)
	IL-1β, IL-2, IL-5, IL-6, IL-8, IL-10, IL-17, IL-12p70, IFN-α, TNF-α	Normal
Tumor Markers	AFP, CEA and NSE	Negative
Pathogen (NGS of BALF)	*Streptococcus pneumoniae*	Positive
	Viral, fungal, tuberculous and parasitic pathogens	Negative
Parasitic antibodies	*Leptospira and Paragonimus*	Negative
Other hematologic workup	coagulation studies, serum iron, TIBC, vitamin B12, direct Coombs test, and hemoglobin electrophoresis	Normal
Bone marrow aspirate		Hypercellular marrow with marked erythroid hyperplasia; nucleated red blood cells exhibited pronounced basophilia, some mature erythrocytes showing central pallor
Imaging	Chest CT	extensive bilateral nodular ground-glass opacities
	Sinus CT	Normal
	Abdominal CT	Normal
	Abdominal ultrasound	Normal
	Echocardiography	Normal
	Brain MRI	Multiple patchy FLAIR hyperintensities and SWI hypointensities in brain parenchyma
	PET-CT	Lung: diffuse, multifocal, small central nodules (SUVmax: 2.7); brain: multiple cortical nodules (normal SUV); right-thigh: a soft tissue mass (SUVmax: 8.8)

ANA: Antinuclear antibody; ds-DNA: Double-stranded deoxyribonucleic acid; ENA: Extractable nuclear antigen; ANCA: Antineutrophil cytoplasmic antibody; CCP: Cyclic citrullinated peptide; RF: Rheumatoid factor; aCL: Anti-cardiolipin; Ig: Immunoglobulin; C3: Complement component 3; C4: Complement component 4; NK: Natural killer cell; IL: Interleukin; IFN: Interferon; AFP: Alpha-fetoprotein; CEA: Carcinoembryonic antigen; NSE: Neuron-specific enolase; NGS: Next-generation sequencing; BALF: Bronchoalveolar lavage fluid; MRI: Magnetic resonance imaging; PET-CT: Positron emission tomography–computed tomography; SUV: Standardized uptake values

The patient was initially treated with intravenous methylprednisolone (2 mg/kg/day), followed by oral prednisone with a gradual taper, ceftriaxone for bacterial infection, and trimethoprim-sulfamethoxazole for Pneumocystis prophylaxis. Despite these interventions, his anemia persisted. Thoracoscopic lung biopsy was performed one month later, when the paitent exhibited mild tachypnea and maintained an oxygen saturation of 98–100%. Histopathology revealed alveolar expansion with associated type II pneumocyte hyperplasia. The alveolar spaces contained abundant hemosiderin-laden macrophages. The alveolar septa exhibited mild thickening with infiltration of scattered lymphocytes and histiocytes. Vascular examination revealed mild degenerative changes in small vessel walls, with no significant perivascular inflammatory cell infiltration. Focal hemorrhagic necrosis and myofibroblast proliferation were observed, accompanied by infiltrating lymphocytes and plasma cells in the surrounding tissue. Immunohistochemical analysis revealed the following staining pattern: AE1/AE3 (positive in epithelial cells), CD20 (positive in scattered B lymphocytes), CD3 (positive in small T lymphocyte aggregates), CD34 (positive in vascular endothelial cells), CD38 (positive in scattered plasma cells), CD68 (KPI) (positive in histiocytes), and SMA (focal positivity in areas of fibrosis) (Fig. 1C, D and E). These findings were consistent with diffuse alveolar hemorrhage syndrome but did not provide definitive clues regarding the underlying etiology.

**Fig. 2:**
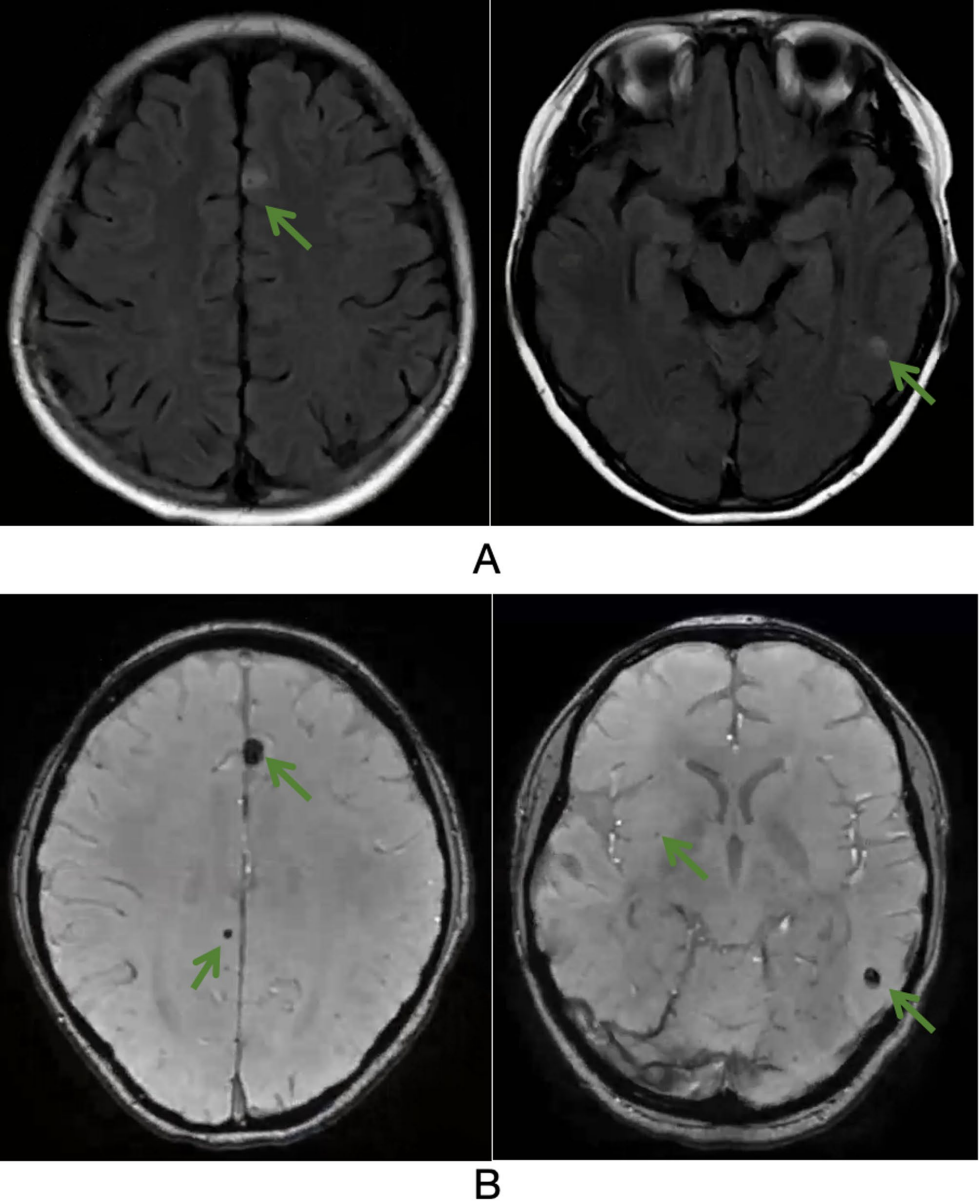
**A**: Brain MRI revealed multiple patchy hyperintensities in the brain parenchyma on FLAIR sequences (indicated by green arrows). **B**: Brain MRI demonstrated multiple scattered punctate and patchy hypointensities in the brain parenchyma on SWI sequences, representing microhemorrhages (indicated by green arrows)

Because children with DAH may develop systemic vasculitic manifestations, brain MRI was obtained to exclude intracranial vasculitis. The investigation revealed unexpected findings: multiple punctate and patchy hyperintensities on fluid-attenuated inversion recovery (FLAIR) sequences, with low T2 and high T1 signals in the bilateral basal ganglia. Susceptibility-weighted imaging (SWI) demonstrated scattered punctate and patchy hypointensities suggestive of microhemorrhages (Fig. 2). Contrast-enhanced brain MRI showed no significant enhancement. Given the concurrent DAH and intracranial hemorrhage, systemic vasculitis was suspected; the patient received high-dose methylprednisolone pulse therapy (10 mg/kg/day for 3 days) followed by a maintenance infusion of 2 mg/kg/day. The response was transient: hemoglobin rose briefly from 65 g/L to 88 g/L within a week, then fell again to 68 g/L by day 15, necessitating repeated packed red blood cell transfusions. A follow-up chest HRCT scan four months later showed persistent bilateral ground-glass opacities.

**Fig. 3:**
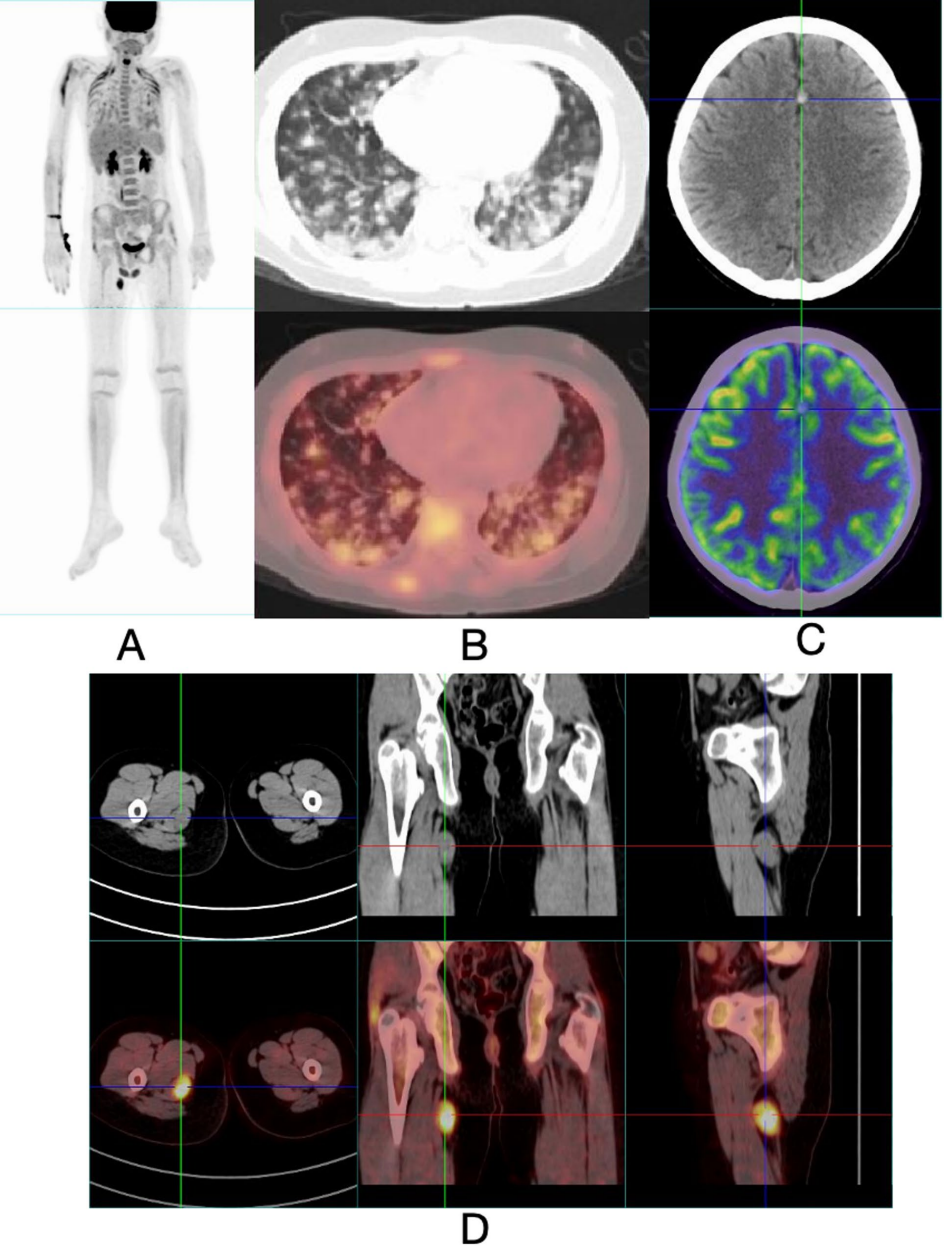
**A**: Maximum intensity projection (MIP) image of whole-body PET-CT. **B**: PET-CT of the lungs demonstrated diffuse, multifocal, small central nodules with moderate FDG uptake (SUVmax: 2.7). The lesions were more prominent in the bilateral lower lobes, accompanied by adjacent pleural thickening and traction, with increased FDG uptake (SUVmax: 1.5). **C**: PET-CT of the brain revealed a cortical nodule in the temporal lobe without abnormal FDG uptake. **D**: PET-CT identified a soft tissue mass (size: 2.6 cm × 1.7 cm × 3.0 cm) in the right thigh with significantly increased FDG uptake (SUVmax: 8.8)

**Fig. 4:**
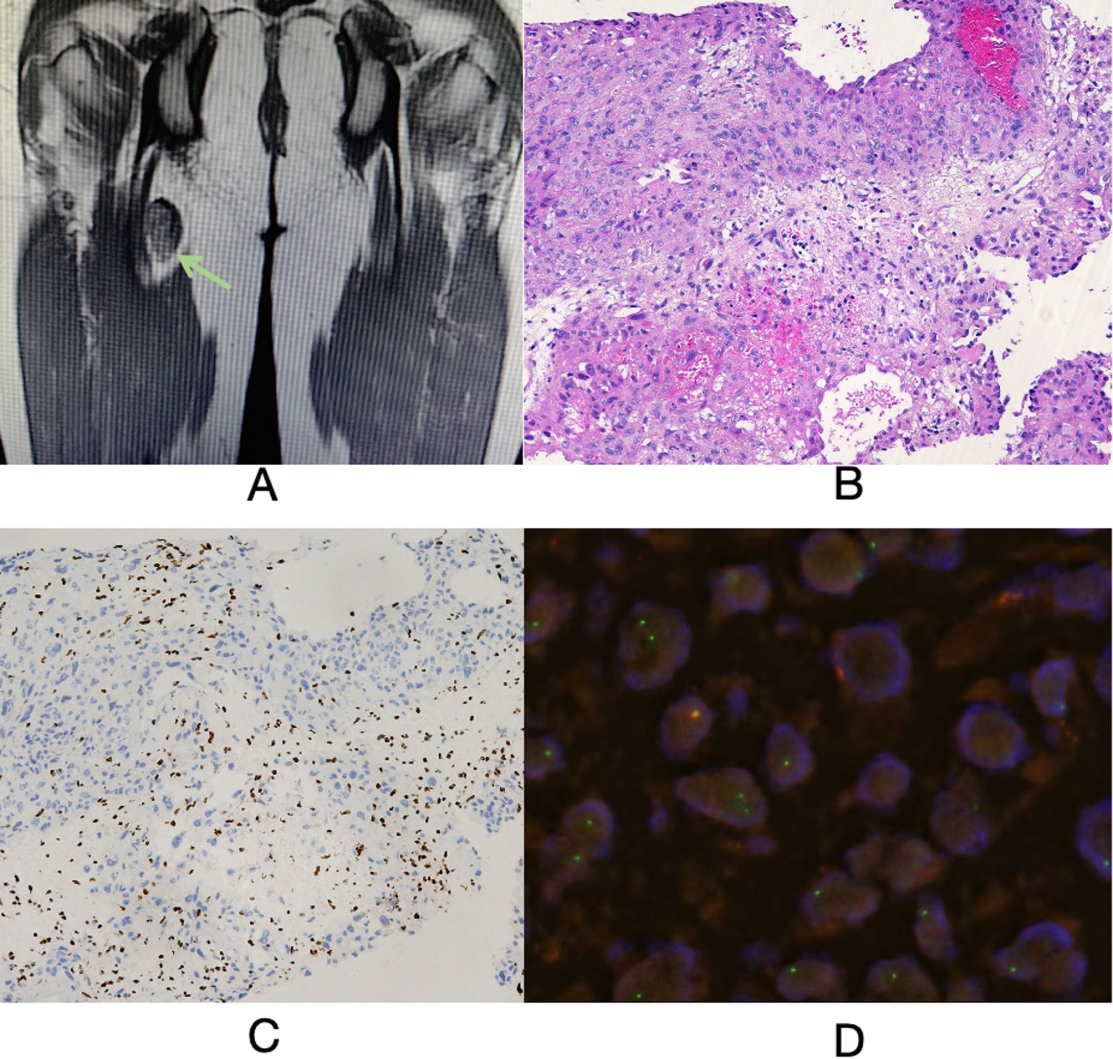
**A**: MRI of the thigh revealed an oval-shaped heterogeneous signal in the posteromedial aspect of the right thigh, with irregular margins and partial blurring of the boundary with the semitendinosus muscle (indicated by the green arrow). **B**: Histopathological analysis of the thigh mass (×100 magnification) demonstrated epithelioid neoplastic cells organized in nested and clustered patterns. The tumor cells exhibited marked cytological atypia, characterized by large cell size, abundant eosinophilic cytoplasm, and round to oval nuclei. Focal nuclear grooves and vesicular chromatin were observed, accompanied by a single prominent, eccentrically located nucleolus. **C**: INI1 immunohistochemical staining of the thigh mass (×100 magnification) revealed loss of INI1 expression. **D**: Fluorescence in situ hybridization (FISH) analysis of the thigh mass (×400 magnification) confirmed SMARCB1/INI1 gene deletion

Given the multisystem involvement and poor response to corticosteroids, a neoplastic process was suspected. PET-CT identified a soft tissue mass (size: 2.6 cm × 1.7 cm × 3.0 cm) in the right thigh with significantly increased FDG uptake (SUVmax: 8.8), highly suggestive of a neoplastic lesion. Additionally, the lungs exhibited diffuse, multifocal, small central nodules with moderate increased FDG uptake (SUVmax: 2.7). These lesions were more prominent in the bilateral lower lobes, accompanied by adjacent pleural thickening and traction, with increased FDG uptake (SUVmax: 1.5). Furthermore, multiple cortical nodules in the left frontal, temporal, and parietal lobes were observed, though they showed no abnormal FDG uptake (Fig. 3). Subsequent MRI of the thigh revealed an oval-shaped heterogeneous signal in the posteromedial aspect of the right thigh, with irregular margins and partial blurring of the boundary with the semitendinosus muscle (Fig. 4A).

Histopathological analysis of the thigh mass demonstrated epithelioid neoplastic cells organized in nested and clustered patterns. The tumor cells displayed marked cytological atypia, characterized by large cell size, abundant eosinophilic cytoplasm, and round to oval nuclei. Focal nuclear grooves and vesicular chromatin were observed, accompanied by a single prominent, eccentrically located nucleolus. Immunohistochemical analysis demonstrated the following profile: INI1 expression was lost (negative), while cytokeratin and Fli-1 were diffusely positive. Focal positivity was noted for CD34, GluT-1, and CD31, with partial expression of ERG. The proliferation index, as assessed by Ki-67, was approximately 15%. Additional markers, including TFE3, synaptophysin, P63, Melan-A, S100, chromogranin A, inhibin-a, HMB45, tyrosine hydroxylase, CAMTA-1, factor VIII, smooth muscle actin, SOX10, and human herpesvirus 8, were uniformly negative. Scant positivity was observed for CD68. These findings supported the diagnosis of an INI1-deficient epithelioid sarcoma (Fig. 4B and C). FISH analysis of the thigh mass identified SMARCB1/INI1 gene deletion, confirming the diagnosis of INI1-deficient epithelioid sarcoma (Fig. 4D).

Unfortunately, after the diagnosis of epithelioid sarcoma, the patient’s parents declined further treatment.

## Discussion

This case presents a rare and intriguing manifestation of INI1-deficient ES characterized by DAH and intracranial hemorrhagic lesions. To our knowledge, this is the first reported case of ES presenting with DAH as the initial clinical feature, highlighting the diagnostic challenges and emphasizing the importance of considering malignancy in pediatric patients with atypical systemic bleeding manifestations.

ES is an exceedingly rare soft tissue sarcoma with a high propensity for recurrence and metastasis. It accounts for only 1% of all soft tissue sarcomas [1]. The incidence rate of ES ranging from 0.03/100,000 to 0.05/100,000, and is very low (0.01/100.000) in childhood population [2]. Histologically, ES is characterized by epithelioid-like tumor cells, typically presenting as slow-growing, painless masses, yet exhibiting aggressive behavior with significant metastatic potential. Metastatic spread is observed in 20–50% of cases during the disease course and approximately 20% of patients present with distant metastases at initial diagnosis [3, 4]. The lungs and lymph nodes are the most frequent sites of metastasis, although distant metastases can also occur in the bones and brain [3]. In addition to pulmonary nodules and consolidation, ES lung metastases may present as cysts, pneumothorax, and pleural effusion [5]. However, no previous reports have documented ES lung metastases associated with diffuse alveolar hemorrhage. In children, ES is classified within the heterogeneous group of non-rhabdomyosarcoma soft tissue sarcomas (NRSTS), constituting about 5% of pediatric NRSTS cases [3]. It predominantly affects older children (7–14 years), with a slight male predominance [3]. The clinical and pathological features of pediatric ES are similar to those in adults, with a predilection for the extremities, particularly the arms and hands [3].

The hallmark molecular alteration in ES is the loss of INI1 (SMARCB1) expression, observed in approximately 90% of cases [6]. SMARCB1, a tumor suppressor gene located at 22q11.2, is a core component of the SWI/SNF chromatin remodeling complex, which regulates gene expression by modulating DNA-histone interactions. The loss of SMARCB1 expression can result from homozygous deletion, bi- or single-allelic deletion, point mutations, or epigenetic silencing [3, 7]. In this patient, FISH analysis confirmed the deletion of the SMARCB1 gene in the thigh mass. Although INI1 deficiency is pathognomonic for ES, it is not exclusive to this entity and can also be observed in a spectrum of other malignancies, including malignant rhabdoid tumors, poorly differentiated chordomas, epithelioid malignant peripheral nerve sheath tumors, epithelioid schwannomas, soft tissue myoepithelial tumors, and SMARCB1-deficient sinonasal carcinoma [2]. Accurate differentiation requires the integration of clinical, immunohistochemical, and radiological findings. In our case, the epithelioid morphology, cytokeratin positivity, and extremity location strongly supported the diagnosis of ES over other INI1-deficient malignancies.

DAH is a rare clinical syndrome characterized by the accumulation of red blood cells within the alveolar spaces. It typically presents as a triad of anemia, hemoptysis, and diffuse ground-glass opacities on chest CT. The diagnosis is often confirmed by the presence of hemosiderin-laden macrophages in BALF cytology. DAH is most commonly associated with autoimmune diseases, particularly systemic vasculitis ans systemic lupus erythematosus. However, it can also result from infections, immune deficiencies, metabolic disorders, coagulopathies, drug toxicity, inhaled toxins, and post-transplantation complications. In some cases, the underlying cause of DAH remains unidentified, leading to a diagnosis of idiopathic pulmonary hemosiderosis (IPH). Malignancy-related DAH is exceedingly rare and may arise from tumor embolism, direct vascular invasion by metastatic tumors, or paraneoplastic immune-mediated mechanisms. For example, pulmonary metastasis of angiosarcoma has been reported in association with DAH [8]. Additionally, paraneoplastic syndromes linked to autoimmune vasculitis, such as those observed in myelodysplastic syndrome, can also lead to DAH [9]. Similarly, pulmonary Kaposi’s sarcoma has been associated with anti-neutrophil cytoplasmic antibody-associated vasculitis and DAH [10, 11].

In this patient, the pathogenesis of ES-induced DAH is likely multifactorial. The lungs are the most frequent site of metastasis in ES. Although no malignant cells were identified in the lung biopsy, the presence of FDG-avid pulmonary nodules on PET-CT supports the hypothesis of tumor metastasis to the lungs. Furthermore, ES-induced paraneoplastic immune-mediated vasculitis may contribute to both DAH and intracranial hemorrhage. Elevated levels of IL-4 and IFN-γ in this case suggest a Th1/Th2 immune imbalance, which may potentially induce immune-mediated vascular injury.

The diagnosis of ES in this case was particularly challenging due to the atypical presentation with DAH and intracranial hemorrhagic lesions. The patient exhibited progressive DAH refractory to corticosteroid therapy, which is inconsistent with autoimmune disease-related DAH and IPH. Infections, coagulopathies, and drug-induced causes were also ruled out. Although malignancy was suspected, both lung biopsy and bone marrow examination failed to reveal any evidence of tumor, further complicating the diagnostic process. The identification of a small tumor in the proximal thigh on PET-CT was pivotal for the final diagnosis, underscoring the critical role of advanced imaging in evaluating unexplained and treatment-refractory DAH.

The primary treatment for ES is radical surgical excision with microscopically clear margins, often combined with perioperative radiotherapy (RT) to reduce the high risk of local recurrence. Adjuvant RT remains a standard approach despite the relative radioresistance of ES [3]. For advanced or metastatic disease, anthracycline-based regimens are the first-line systemic therapy, while gemcitabine-based therapies have shown particular efficacy in distal-type ES [12, 13]. Emerging targeted therapies, such as the enhancer of zeste homolog 2 (EZH2) inhibitor tazemetostat, demonstrate promising clinical activity and may provide new therapeutic options [2, 14]. The prognosis for patients with ES is generally poor, with a five-year overall survival rate of approximately 50% [2]. In children, adolescents, and young adults, the five-year overall survival rate was 48%, dropping to a dismal 9% in patients presenting with metastatic disease [15]. These findings highlight the aggressive nature of ES and emphasize the importance of early diagnosis.

## Conclusion

This case highlights the importance of considering malignancy in pediatric patients presenting with DAH, particularly when accompanied by multisystem involvement and poor response to corticosteroid treatment. A multidisciplinary approach, incorporating advanced imaging techniques such as PET-CT and molecular diagnostics, is crucial for the accurate diagnosis and management of rare malignancies like ES. Early recognition and the use of targeted therapies may improve clinical outcomes in such challenging cases.

## Data Availability

The datasets generated and analyzed during this study are not publicly available due to patient privacy and confidentiality concerns. However, anonymized data can be provided by the corresponding author upon reasonable request.

## Abbreviations

BALF: Bronchoalveolar lavage fluid
DAH: Diffuse alveolar hemorrhage
ES: Epithelioid sarcoma
EZH2: Enhancer of zeste homolog 2
FDG: Fluorodeoxyglucose
FISH: Fluorescence in situ hybridization
FLAIR: Fluid-attenuated inversion recovery
HRCT: High-resolution chest CT
IFN: Interferon
Ig: Immunoglobulin
IL: Interleukin
IPH: Idiopathic pulmonary hemosiderosis
NRSTS: Non-rhabdomyosarcoma soft tissue sarcomas
PET-CT: Positron emission tomography - computed tomography
RT: Perioperative radiotherapy
SUV: Standardized uptake values
SWI: Susceptibility-weighted imaging
Th: T-helper lymphocytes
